# Is It Time to Call Time on Bone Marrow Biopsy for Staging Ewing Sarcoma (ES)?

**DOI:** 10.3390/cancers13133261

**Published:** 2021-06-29

**Authors:** Katrina M. Ingley, Simon Wan, Stefan Vöö, Rachael Windsor, Maria Michelagnoli, Asif Saifuddin, Sandra J. Strauss

**Affiliations:** 1 The London Sarcoma Service, University College London Hospitals Foundation Trusts, London NW1 2BU, UK; katrina.ingley@nhs.net (K.M.I.); rachael.windsor@nhs.net (R.W.); maria.michelagnoli@nhs.net (M.M.); 2 Institute of Nuclear Medicine, University College London Hospital NHS Foundation Trust, London NW1 2BU, UK; mwan@nhs.net (S.W.); stefan.voo@nhs.net (S.V.); 3 Biomedical Research Centre, Inflammation, Immunity and Immunotherapeutics, University College London Hospital, London NW1 2BU, UK; 4 Department of Radiology, The Royal National Orthopaedic Hospital NHS Trust, Brockley Hill, Stanmore, London HA7 4LP, UK; asif.saifuddin@nhs.net; 5 UCL Cancer Institute, University College, London WC1E 6DD, UK

**Keywords:** Ewing sarcoma, staging, bone/bone marrow metastases, bone marrow biopsy, 18F-fluorodeoxyglucose (18FDG), positron emission tomography/computed tomography (PET/CT), functional imaging, whole body magnetic resonance imaging (WB-MRI), bone scintigraphy

## Abstract

**Simple Summary:**

Ewing sarcoma (ES) is a rare primary bone cancer, usually found in children and adolescents, which can spread to the lungs, other bones and less commonly, the bone marrow. An accurate determination of the disease spread at baseline (staging) is important to establish prognosis, monitor treatment response and help with management decisions. There is no standard of care for staging ES, although the invasive bone marrow biopsy has traditionally been used to establish whether patients have bone marrow infiltration. Imaging techniques, including FDG-PET/CT and whole-body MRI (WB-MRI), have become established in staging other cancers with expanding use for staging ES. A number of studies have validated the accuracy, sensitivity and specificity of these modalities for detecting bone and bone marrow metastases in ES. The main aim of this review was to examine the current literature for the use of FDG-PET/CT and WB-MRI in staging ES to determine whether a bone marrow biopsy is still needed and would influence the management of patients. Hereafter, a new staging algorithm for ES recommends WB-MRI and/or FDG-PET/CT without bone marrow biopsy as the standard of care for staging localised and metastatic ES.

**Abstract:**

Primary malignant bone sarcomas are rare and Ewing sarcoma (ES), along with osteosarcoma, predominates in teenagers and young adults. The well-established multimodality treatment incorporates systemic chemotherapy with local control in the form of surgery, with or without radiation. The presence and extent of metastases at diagnosis remains the most important prognostic factor in determining patient outcome; patients with skeletal metastases or bone marrow infiltration having a significantly worse outcome than those with lung metastases alone. There is, however, no accepted staging algorithm for ES. Large cooperative groups and national guidelines continue to advocate bone marrow biopsy (BMB) for staging but functional imaging techniques, such as 18F-fluorodeoxyglucose positron emission tomography (FDG-PET) with computerised tomography (CT) have been increasingly used for staging cancers and whole-body magnetic resonance imaging (WB-MRI) for staging skeletal metastases. This review outlines the current literature, from which we conclude that BMB is no longer required for the staging of ES as it does not influence the standard of care management. BMB may, however, provide prognostic information and insights into the biology of ES in selected patients on prospective clinical trials.

## 1. Introduction

Ewing sarcoma (ES) is the second most common primary bone malignancy among children, teenagers and young adults (TYA) [1]. Accurate staging of patients is important for the determination of the extent of disease to optimise treatment and establish prognosis. The traditional staging of ES included chest computed tomography (CT) to identify lung metastases, a technetium bone scan to identify bone metastases and a bilateral bone marrow biopsy (BMB) to identify bone marrow involvement. BMB is, however, an invasive procedure that conveys a small risk of complications, and is limited by focal variation in sampling [2].

Over recent decades, imaging techniques such as 18F-fluorodeoxyglucose positron emission tomography with computerised tomography (FDG-PET/CT) and/or whole body magnetic resonance imaging (WB-MRI) have been developed that are increasingly used to stage patients with solid tumours, including ES. Outstanding questions remain on the appropriate role of these techniques in the clinical management of patients with ES. One such question is whether noninvasive FDG-PET/CT and WB-MRI can circumvent the need for BMB as standard staging in ES.

This article focuses on the use of FDG-PET/CT and/or WB-MRI in the staging of ES patients at diagnosis, with an emphasis on the review of comparative literature between imaging and BMB.

## 2. Prognosis and Management of ES

Approximately 25% of patients with ES have detectable metastases at diagnosis with conventional staging methods. Metastases are predominantly located in the lungs (38%), bone (31%) and bone marrow (11%) [1]. Patients with localised disease have a 5-year overall survival (OS) of 65–75% compared to approximately 30% for those with metastatic disease [3]. Patients with isolated lung metastases have a 5-year OS reaching 50%, compared to those with bone and bone marrow disease who have a significantly worse outcome [3,4]. For those with widespread bone disease, a 5-year event free survival (EFS) is approximately 16% and less than 5% if bone marrow metastases are present [4,5].

The management of ES involves multimodality treatment with intensive multiagent chemotherapeutic agents and local control involving surgery and/or radiation therapy (RT). The current standard of care incorporates interval-compressed vincristine, doxorubicin, cyclophosphamide, alternating with ifosfamide, etoposide (VDC-IE) with a 5 year EFS of 73% and OS of 83% across all patients [6,7]. Despite the poor outcome of patients with metastatic disease, patients receive the same first line therapy. High dose (HD) chemotherapy and autologous stem cell transplant (ASCT) have been investigated for selected patients [8,9], however, for patients with widely disseminated disease, their role remains controversial, and outcomes poor.

Imaging techniques that enable the accurate detection of the number and location of metastases play an important role in the management of patients with ES in whom the extent of disease impacts on outcome. In patients with oligometastatic bone disease, (usually defined as 1 to 5 lesions), locally ablative treatments are considered to improve survival; this survival benefit persisting with repeated local treatments for recurrence of oligometastases [10].

## 3. FDG-PET/CT and WB-MRI Staging in ES

Functional imaging using FDG-PET/CT identifies the upregulated glucose metabolism in tumours and has many applications in oncology [11,12,13,14,15,16,17,18]. For assessment of the primary tumour in ES, baseline and post treatment quantitative FDG-PET/CT parameters, such as standardised uptake values (SUV), metabolic tumour volume and total lesion glycolysis, have been explored in the neoadjuvant treatment setting and are found to correlate with histological response [19,20]. FDG-PET/CT has been reported as potentially useful, but an inconsistent predictor of survival outcomes. Its role in response assessment requires evaluation in prospective studies [21,22].

FDG-PET/CT imaging has shown high sensitivity, specificity and accuracy for detecting distant metastases in staging ES [23,24,25]. A large paediatric series of 314 lesions from primary bone sarcomas reported FDG-PET/CT to be the superior imaging modality to detect all metastatic sites excluding the lungs, with a sensitivity of 83% and specificity 98% versus 78 and 97% for conventional imaging (CT, ultrasound, MRI and/or bone scintigraphy) [26]. The authors used histopathology or further imaging combined with at least 6 months follow-up to confirm the lesion status [26]. Metabolic characterisation of small lung lesions can be limited due to low spatial resolution for lesions < 0.7 cm that underestimates FDG uptake and lung nodules close to mediastinum and lung bases are not detected well, thus high-resolution CT scanning is recommended to detect lung metastases [27].

In a meta-analysis of 23 studies, Huang et al. demonstrated high diagnostic accuracy for FDG-PET/CT with sensitivity and specificity of 86 and 80% for detecting distant metastases from 13 studies involving 689 lesions [28]. This included 544 patients; 507 patients from 20 retrospective studies, 31 patients from 2 prospective studies and 6 patients from 1 study where the design was not reported. The studies had small patient populations with an average of 24 patients per study with different techniques utilised for measuring FDG uptake and with differing reference standards [28]. Six studies specifically evaluated bone metastases using FDG-PET/CT and demonstrated a high sensitivity of 91% and specificity of 98% in the 188 patients evaluated [28]. Volker et al. demonstrated FDG-PET/CT was superior for the detection of lymph node and bone involvement compared to conventional imaging by prospective analyses in a paediatric and adolescent sarcoma population of 46 (ES cohort, *n* = 23) [27]. The sensitivity for detecting additional bone metastases in ES was significantly higher with FDG-PET/CT over conventional imaging, 88 vs. 37% (*p* < 0.01) [27].

WB-MRI enables the examination of the entire body with excellent contrast and spatial resolution without exposure to ionising radiation [29]. Several studies have demonstrated the value of WB-MRI for detecting bone metastases in a variety of solid cancers [30,31] and large prospective multicentre trials within England have demonstrated comparability to conventional staging pathways that incorporated PET-CT in non-small cell lung cancer and colorectal cancer [32,33,34]. A systematic review investigating the diagnostic performance of WB-MRI included five relevant studies, totalling 132 patients < 19 years age, 39 (40.6%) with a diagnosis of ES demonstrating a sensitivity of WB-MRI for detecting bone metastases ranged between 82 and 100% [35]. The studies included heterogeneous cohorts of children with various primary solid tumours using a variable reference standard leading to unclear or high risk of bias [35].

WB-MRI and FDG-PET/CT were significantly superior to bone scintigraphy in detecting bone metastases in a paediatric population that included 11 patients with ES [36]. The sensitivity and specificity were 97.5 and 99.4% for WB-MRI, 90 and 100% for FDG-PET/CT and 30 and 99.4% for bone scintigraphy, respectively [36]. WB-MRI and FDG-PET/CT demonstrated excellent concordance with the final diagnosis, 96.9 and 93.6%, respectively [36]. The two imaging modalities were equally effective, revealing the same lesions in most body regions, except WB-MRI detected more spinal lesions [36]. A recent prospective diagnostic study on primary bone sarcoma including ES (*n* = 30) used frequency tables and multidisciplinary team consensus to compare the staging of bone metastases by WB-MRI and/or FDG-PET/CT with bone scan [37]. The sensitivity was 88, 88, 50%, specificity 95, 100, 95%, PPV, 88, 100, 80% and NNV, 95, 96, 84% for WB-MRI, FDG-PET/CT and bone scan, respectively [37].

Comparative retrospective studies have corroborated the superiority of FDG-PET and WB-MRI over bone scintigraphy in detecting bone metastases in ES with the exception of skull vault lesions discerned by bone scinitigraphy [38,39,40]. In a 12-year retrospective review comprising 182 ES patients, Kalus et al. reported a greater number of bone metastases were detected through staging with WB-MRI compared with bone scan, 24 and 16.9%, respectively [40]. In 13 patients (18.3%) who had both modalities performed and bone metastases detected, 4 had bone metastases only identified on WB-MRI [40].

Daldrup-Link et al. compared FDG-PET/CT with WB-MRI in 39 paediatric and adolescent patients with mixed solid tumours, 20 with ES [23]. FDG-PET/CT had a superior sensitivity, 90 versus 82% for WB-MRI and 71% for bone scan (*p* < 0.05) [23]. False positive lesions were more frequent with FDG-PET/CT in 2/39, compared with 0/39 by WB-MRI and bone scan due to a number of inflammatory conditions and physiological uptake that can induce glycolytic activity. Whereas false negative lesions occurred more commonly with WB-MRI (usually in small or flat bones such as the ribs) and bone scan (spinal lesions), in 5/39 patients each, compared with 3 patients by FDG-PET/CT (skull lesions) [23].

In a small retrospective study, the authors identified 112 bone lesions in 13 of 20 patients with ES [41]. Sensitivity and specificity by lesion were 62 and 100% for FDG-PET/CT and 99 and 100% for WB-MRI. Although WB-MRI detected a higher number of skeletal lesions, with FDG-PET/CT no patients with metastatic disease were missed and in 12 of 13 patients (92.3%) FDG-PET/CT and MRI were concordantly positive for bone metastases [41]. PET and MRI were both negative for bone metastases in 7 of 20 patients [41]. The number of skeletal metastases was under-represented by FDG-PET/CT in a small number of patients compared with MRI. False negatives were more likely to occur in patients with small lesions, <10 mm in the axial skeleton and when there was widespread active red bone marrow, such as from recent chemotherapy [41].

**Figure 1:**
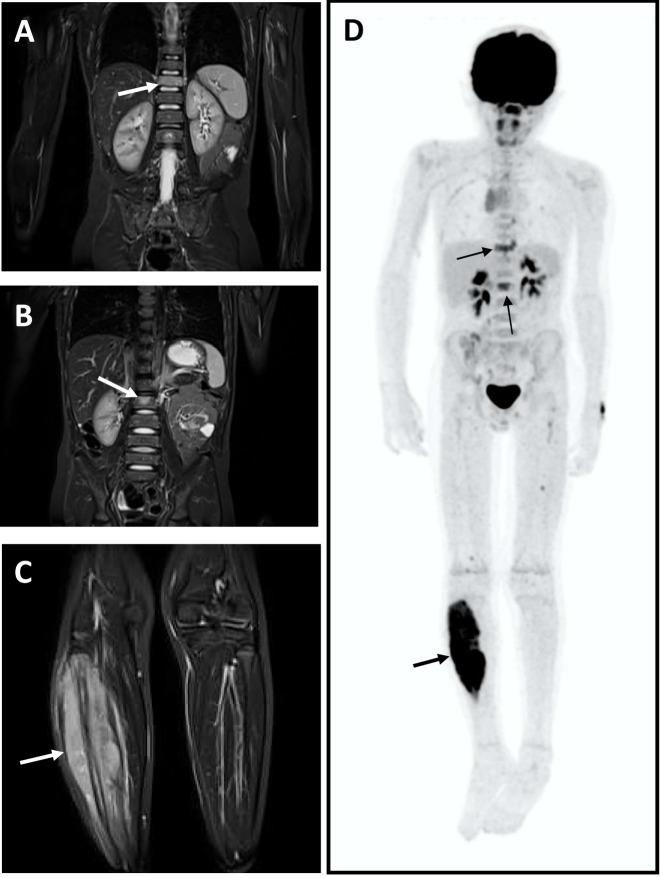
Nine-year-old boy with primary Ewing sarcoma of the right fibula and spinal bone metastases (**A**–**C**). Coronal STIR MR images from a whole-body MRI study show the primary tumour (arrow—**C**) and metastases in the spine (arrows—**A**,**B**). Coronal PET study demonstrates intense FDG uptake in the right fibula (**D**) (thick arrow) and also in the spinal metastases (thin arrows).

In summary, FDG-PET/CT has been shown to be accurate in the diagnosis of metastatic disease in ES, particularly in the identification of bone metastases (Figure 1). WB-MRI also appears superior to bone scintigraphy and comparable to FDG-PET/CT and has the advantage of not requiring ionising radiation. A theoretical limitation of the available studies is the lack of an independent reference standard for the precise number of true metastatic deposits. Outcome studies involving the use of these different imaging modalities to direct treatment remains lacking.

## 4. FDG-PET/CT Detection of BM Metastases

A strong relationship exists between the presence of bone metastases identified on imaging at staging and primary involvement of the bone marrow. This premise underlines the design of studies attempting to compare the effectiveness of functional imaging to detect bone marrow involvement (Table 1). The majority of studies in Table 1 define FDG-PET/CT positivity with the presence of one or more osseous metastatic lesions per patient. The correlation between the findings of the BMB and FDG-PET/CT are therefore through association with the presence of osseous metastatic disease or not, and not specifically imaging changes at the BMB sites. These studies propose FDG-PET/CT imaging allows for accurate bone marrow staging in ES.

A retrospective study of 20 patients with newly diagnosed ES underwent staging FDG-PET/CT with a total of 38 BMB [45]. FDG-PET/CT and BMB were concordantly positive in 3 patients and negative in 16 patients, with agreement between the modalities at 95% at a patient level. This is a unique study in that the authors also reported specifically whether there were positive FDG-PET/CT observations at the posterior ilium where BMBs were performed. FDG-PET/CT and BMB were concordant in 36 of 38 BMB sites (94.7%). The two sites with discrepant FDG-PET/CT and BMB findings were reported in the same patient, in which the FDG-PET/CT was consistent with widespread bone marrow disease and the 2 BMBs were negative [45].

Another study reviewed 26 patients diagnosed with ES who underwent BMB and FDG-PET/CT at staging [44]. All 21 patients with localised disease on FDG-PET/CT had a clear BMB. The sensitivity of bone marrow involvement for patients in which FDG-PET/CT also ascertained bone metastases was 75% (3/4) and 100% specificity (22/22) [44]. The 3 patients with a positive BMB demonstrated bone metastases by FDG-PET/CT as well [44].

Oberlin et al. previously upheld the value of BMB in staging and reported bone marrow involvement in 13/59 (22%) of ES patients and in 52% of those patients with metastases at other sites, with a high correlation in those that also had bone metastases detected by bone scan, and less association in those with lung metastases, 3/10 (30%) [48]. A retrospective analysis of a large cohort of patients (*n* = 504) with ES demonstrated 137 (27%) had metastases at diagnosis by initial imaging using a combination of different modalities, and 12 (2.4%) had a positive BMB [42]. The incidence of patients with bone marrow infiltration was 11/136 (8%) in patients with distant metastases on imaging and 11 of 12 patients with BMB positivity had synchronous bone metastases on FDG-PET/CT or bone scan only. One patient out of 368 (0.3%) had confirmed bone marrow involvement on BMB not detected on imaging, however the patient was investigated with a bone scan only [42]. Comparably, in a multi-institutional retrospective review of 116 patients with ES, of those with metastatic disease on imaging, 42% had a positive BMB [47]. Patients with a pelvic primary rather than a nonpelvic primary were more likely to have bone marrow involvement, 21% versus 9% [47]. Osseous metastases discovered on imaging had a significantly higher correlation with BMB positivity (*p* = 0.002), compared to other metastatic sites such as the lung (*p* = 0.017) [47]. Importantly, none of the 85 patients considered non-metastatic by combination imaging at staging, including MRI, whole body bone scans and chest CT, were BMB positive [47].

A large retrospective study of 91 patients with ES, diagnosed between 2001 and 2011 compared staging FDG-PET/CT and bone scan for evaluating osseous metastatic disease, defined as the presence of at least one distant bone metastasis [25]. There was a high concordance rate of 98% between the two imaging techniques. Similarly, the authors found that bone scan and FDG-PET/CT were highly predictive of bone marrow metastases. From 69 patients who had both FDG-PET/CT and a BMB, all 6 patients with bone marrow metastases also had osseous metastatic disease recognised by FDG-PET/CT and bone scan [25]. There were no bone marrow metastases discovered on any patients that were also negative by bone scan and FDG-PET/CT [25]. Thus, the authors suggest that FDG-PET/CT may suffice for staging bone metastases and bone marrow sampling may only be indicated if osseous metastatic disease is detected by imaging modalities at diagnosis. There are no studies that directly compare WB-MRI and/ or FDG-PET/CT and BMB in ES for the detection of bone marrow metastases.

Other recent studies have questioned the utility of BMB for staging ES [49,50]. In a systematic review that included data on patients from 38 studies with newly diagnosed ES, the pooled incidence of bone marrow metastases was 4.8% in newly diagnosed ES and 17.5% in patients with metastatic disease [49]. In four select studies that used FDG-PET/CT for staging newly diagnosed ES comprising 142 patients [25,43,44,45] the sensitivity, specificity, PPV and NPV of FDG-PET/CT to detect bone marrow metastases compared with BMB was 100, 96, 75 and 100%, respectively [49]. The same review found 18/1663 (1.1%) of patients with newly diagnosed ES presented with isolated bone marrow metastases. In all of these reported cases in which staging workup was detailed, a bone scan only was used for staging and potentially could have missed other distant bone metastases.

The observation of a strong association between bone metastases diagnosed on FDG-PET/CT and bone marrow infiltration on BMB is not unexpected. It has been postulated that bone metastases develop as a multistep process, commencing with metastatic seeding in the vascularised marrow, followed over time with destructive or reactive change in mineralised bone later in the process [51,52,53]. FDG-PET/CT can detect this pathophysiology early, being able to probe tumoural activity in the marrow directly, rather than detecting late secondary changes, as is the case for CT, plain radiography and bone scintigraphy. It is therefore probable that FDG-PET/CT and BMB are both surrogate markers for the assessment of tumour load in an individual patient’s bone marrow compartment: with BMB sensitive at the cellular and tissue level, but subject to sampling error limited to the specific anatomical site where the trephine is taken; and with FDG-PET/CT being able to assess ‘macroscopic’ marrow deposits throughout the body, though limited by spatial and contrast resolution.

Indirectly corroborating with the above postulate is the relative lack of similar strong association between lung metastases and BMB positivity, despite the lung being the most common site of ES dissemination. This infers that the association seen between FDG-PET/CT defined skeletal metastases and BMB positivity is not one through an indirect relationship via ‘overall metastatic burden’ in any given patient. Of the few studies reporting results for pulmonary metastases in addition to BMB results in patients with ES, Kopp et al. reported a lower association of lung metastases with bone marrow positivity [47]. In 13 patients with BMB positivity, 6 had lung metastases and of 18 patients having negative BMB, 16 had lung metastases [47]. Similarly in Newman’s cohort, while all 6 BMB positive cases had positive FDG-PET/CT for osseous metastases, it would appear that in 12 patients with definite pulmonary metastases seen on FDG-PET/CT, 10 had a negative BMB and only 2 had both pulmonary metastases and BMB positivity [25]. Cesari et al. noted 5 patients had lung metastases detected with imaging assessment in the 12 patients with known bone marrow involvement, 11 of which had bone metastases on imaging [42]. In the systematic review by Campbell et al., from 6 relevant studies they reported 8.8% (20/225) of ES patients with lung metastases also had bone marrow metastases without bone metastases [49]. However, there is a caveat that not all of these studies from earlier years had imaging techniques available as standard for the modern days, including FDG-PET/CT or WB-MRI, additional distant metastatic sites could have remained undiagnosed and underestimated.

## 5. Current International Guidelines

International guideline recommendations for BMB staging in ES are not consistent, with some offering alternative staging algorithms for bone marrow involvement, whereas others incorporate BMB for definitive ES staging. The Children’s Oncology Group (COG) reports that functional imaging with FDG-PET/CT, preferably prior to diagnostic biopsy, appears superior to scintigraphy for detecting bone metastases at diagnosis [54]. Current COG trials (AEWS1221) utilise FDG-PET/CT to confirm bone metastases and there remains a requirement for bilateral bone marrow biopsies. The UK bone sarcoma guidelines, published in 2016, state that WB-MRI and FDG-PET/CT may be considered for ES staging [55]. In light of the retrospective trial by Newman et al., the application of FDG-PET/CT for bone metastases is supported and considered useful for detecting bone marrow involvement [25]. However, the guidelines specify ‘bone marrow biopsy should be routinely performed as a staging investigation’ [55]. The Euro Ewing 2012 study recommended a staging BMB and if metastases are detected, follow-up bone marrow biopsies to assess response and progression. Conversely, the National Comprehensive Cancer Network (NCCN) guidelines for bone cancer, version 2.2017 suggest a screening MRI of the spine and pelvis can be considered instead of BMB [56]. Likewise, the European Society for Medical Oncology (ESMO) 2018 guidelines refer to the Newman study [25] and comment, ‘there is a very low incidence of bone marrow metastases in localised disease if the PET scan is negative.’ Nevertheless, the guidelines incline towards BMB requirement for staging [57].

## 6. Recommendations

**Figure 2:**
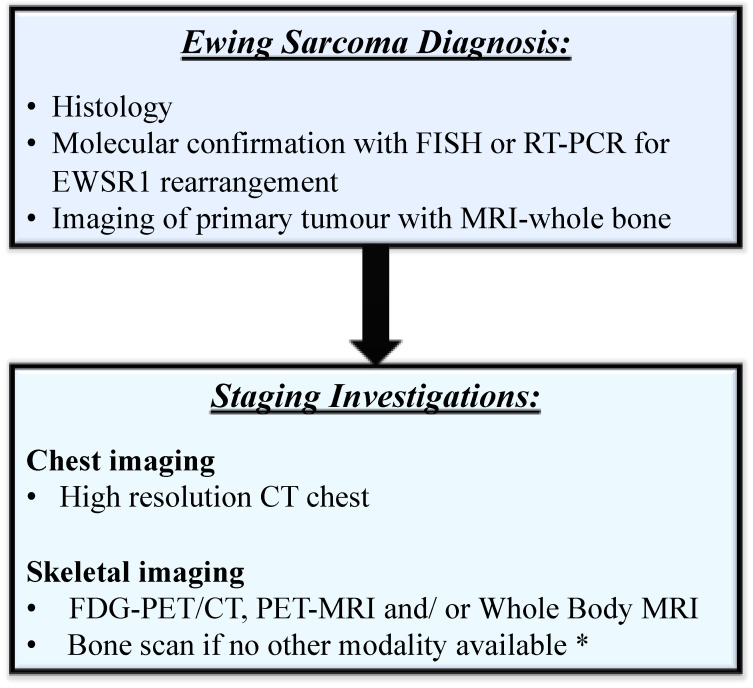
Staging algorithm for patients with Ewing sarcoma undergoing standard of care treatment. * If bone scan only available, consider bone marrow: patients with oligometastatic disease being considered for radical local therapy to all sites. FDG-PET = fluorodeoxyglucose positron emission tomography, WB-MRI = whole body magnetic resonance imaging.

Published guidelines recognise the value and practicality of modern imaging techniques and acknowledge expert opinions. Nevertheless, due to lack of prospective conclusive data, large international trial groups continue to include BMB in standard staging for ES. The presence of bone metastases by FDG-PET/CT is however, strongly associated with bone marrow involvement. There is evidence from institutional series that functional imaging by FDG-PET/CT and WB-MRI is superior to conventional imaging for the detection of bone metastases. Retrospective analyses largely conducted on the paediatric and TYA population have shown within their limits that FDG-PET/CT has excellent sensitivity for bone metastases and is equivalent to BMB sampling for localised disease (at the per patient level). The bone scan has lower sensitivity [23,40], thus if it is the only modality available for skeletal imaging, BMB could be considered in specific circumstances, such as for patients with oligometastatic disease being considered for radiation to all sites. In distinguishing localised from metastatic disease, including bone marrow involvement, the performance of a staging BMB does not add to the sensitivity accomplished with noninvasive modern-day functional imaging. A similar paradigm shift has been adopted in other tumour groups where bone marrow involvement has been identified on functional imaging, such as Hodgkin lymphoma, diffuse large B-cell lymphoma (DLBCL) and small cell lung cancer (SCLC) [58,59]. A recent systematic review also supports functional imaging with FDG-PET/CT for staging newly diagnosed ES patients and omits the BMB in localised disease [49]. We are in agreement with this staging strategy, but further propose that on the basis of modern imaging using FDG-PET/CT and/or WB-MRI that a BMB is also not performed in patients with metastatic disease. If bone scan is the only modality available for evaluating distant metastases, we recommend BMB only if it will potentially change patient management. We have proposed an imaging algorithm for staging ES that reflects the preferable use of FDG-PET/CT and/or WB-MRI for the evaluation of distant metastases, including the bone marrow. We recommend BMB evaluation is omitted from routine staging for localised and metastatic ES disease in patients undergoing standard of care management outside of clinical trials (Figure 2).

## 7. Limitations

The majority of studies that compared FDG PET/CT to BMB are retrospective in nature, single centre and restricted by small cohort size which may limit generalisability of the data and may have led to patient selection bias. In addition, for definitive comparison of both these imaging modalities with BMB, histopathological confirmation of all suspected metastatic sites is required, which is challenging in the clinical setting and was not performed. As the diagnosis and classification of ES has evolved in recent years with the increased application of molecular pathology, studies may have included ‘Ewing-like’ sarcoma subtypes, which have a different natural history, but are unlikely to have influenced the conclusions. Finally, our proposed staging algorithm by omitting BMB in patients with metastatic ES, potentially provides less data to inform prognosis for a group with particularly poor outcome. However, after critical appraisal of the literature, we have taken a pragmatic stance that is consistent with conclusions drawn by several studies, and allows for a consensus approach for the management of ES patients within a specialised sarcoma expert multidisciplinary setting. A cost effectiveness analysis between FDG-PET/CT, WB-MRI and BMAB has not been performed and this is outside the scope of our review.

## 8. Conclusions

To date, a consensus on the inclusion of bone marrow biopsy in standardised staging of ES has not been reached. Based on a review of the current literature, we conclude that there is no additional value in performing a BMB in patients who undergo FDG-PET/CT or WB-MRI as it does not alter the standard of care management. The potential value and information obtained from distinguishing definitive bone marrow involvement through a staging BMB in metastatic ES is currently restricted to providing prognostic information. BMB may continue to be of value in prospective clinical trials that incorporate large homogenous cohorts of patients with ES, to prospectively compare and validate imaging findings and to investigate novel biomarkers.

## Figures and Tables

**Table 1:** Retrospective studies comparing imaging modalities to bone marrow biopsy (BMB) in previously untreated ES.

Ref.	Pt No.	Patient Group	Anatomic Imaging	Functional Imaging	BMB	Outcomes
Cesari [42]	504	ES of bone Median age 16 y (1–68 y) 1998–2017	Chest CT (*n* = 504), bone scintigraphy (*n* = 366), WB MRI (*n* = 1), WB CT (*n* = 1)	FDG-PET/CT (*n* = 130) Bone scan and FDG-PET/CT (*n* = 6)	BMAB (*n* = 504, unilateral)	▪ 137 (27%) metastatic, (30% bone/BM metastases) ▪ 2.4% incidence of +BMB ▪ 8% incidence of +BMAB in patients with bone metastases on imaging ▪ One patient with ES of the metatarsus (1/368, 0.3%) with no distant metastases on imaging (bone scan only) had BM involvement
Yagci-Kupeli [43]	94	94 solid tumours (ES *n* = 16) Median age 12 y (1–18 y) 2014–2017		FDG-PET/CT	BMB	▪ ES: 4/16 had BM involvement by PET; negative BMB in 3 of 4 ▪ ES: sensitivity and specificity of FDG-PET/CT for BM metastases was 100% each and 75% and 8.3% for BMB
Inagaki [44]	26	ES Median age 26 y (11–53 y) 2010–2016	CT contrast (*n* = 25), MRI primary (*n* = 11)	FDG-PET/CT (*n* = 26)	BMAB (*n* = 26, unilateral)	▪ Localised disease by PET (*n* = 21), all BMAB negative ▪ 5 patients PET+ metastatic disease, 3 (60%) +BMAB ▪ 75% (3/4) sensitivity and 100% (22/22) specificity of BM involvement if bone metastases detected by PET
Kasalak [45]	20	ES Mean age 15.9 y (5–57 y) 2009–2017		FDG-PET/CT (*n* = 20)	BMB (18 bilateral, 2 unilateral)	▪ FDG-PET/CT and BMB concordant in 19 of 20 patients (1 patient PET+ and bilateral BMB’s negative)
Zapata [46]	69	69 mixed solid tumours ES (*n* = 7): mean age 10.7 y (3–16 y) 2009–2014		FDG-PET/CT (*n* = 69)-reported on presence of bone marrow disease	BMAB	▪ ES: 4 PET and BMAB negative, 1 patient PET and BMAB+ ▪ 2 PET + patients were BMAB negative
Kopp [47]	116	ES Median age 13 y (1–38 y) 2000–2012	Chest CT, MRI primary	Bone scans	BMAB (*n* = 111/116, bilateral)	▪ 31 metastatic and 85 localised ES ▪ 0/85 with localised ES by imaging had +BMA/B ▪ 13/31 (42%) patients with metastases had +BMAB ▪ Bone metastases by bone scan (*n* = 16) highly correlated with +BMAB (*n* = 12) (*p* = 0.0002)
Newman [25]	91	ES Median age 14.9 y (3.8–56.2 y) 2001–2011		FDG-PET/CT (*n* = 80) Bone scan (*n* = 74)	BMA/B (*n* = 80 patients: 59 aspirates, 62 bilateral biopsies)	▪ Imaging concordance rate between PET and bone scan = 98% (1 patient +PET and negative bone scan) ▪ 0/57 patients without bone metastases on FDG-PET/CT had BM involvement ▪ 6/6 patients with BM metastases had bone metastases by PET and bone scan

Definitions: ES = Ewing sarcoma, BM = bone marrow, BMAB = bone marrow aspirate biopsy, y = year.

## Data Availability

Not applicable.
